# Orthostatic stress echocardiography as a useful test to measure variability of transvalvular pressure gradients in aortic stenosis

**DOI:** 10.1186/1476-7120-11-15

**Published:** 2013-05-24

**Authors:** Pawel Petkow Dimitrow, Danuta Sorysz

**Affiliations:** 1 2nd Department of Cardiology, CMUJ, Kopernika 17, Krakow 31-501, Poland

**Keywords:** Aortic stenosis, Orthostatic stress test

## Abstract

The aim of the study was to assess the influence of the orthostatic stress test on changes in aortic pressure gradients in patients with aortic stenosis (AS).

**Methods:**

The orthostatic stress test was performed in 56 AS patients. The maximum aortic gradient was compared between the supine and the upright position (using Doppler echocardiography from the apical window). The left hand of each patient was kept on top of their head for both readings. 21 patients were excluded from the study for three reasons: 1) atrial fibrillation (significant beat-to-beat variability of measured gradient), 2) suboptimal Doppler signal during the orthostatic test, and 3) aortic gradient significantly higher in suprasternal or right parasternal windows than in apical window (different direction of stenosed blood jets) in the supine examination. The last limitation (#3) is methodologically important because during the orthostatic examination, only the transapical measurement was used. We were able to analyze 35 AS patients (20 males, 15 females, mean age 74.8 ± 9.2 years).

**Results:**

The wide range of severity of AS was examined (maximal aortic gradient in the supine position from 30 to 146 mmHg). With regard to statistical trends, the mean value of the maximum aortic gradient significantly decreased after orthostatic stress (from 87.5 ± 28.6 to 75.8 ± 23.7 mmHg), p > 0.01). In 7 patients (increasing responders) the peak aortic gradient slightly increased during the stress test. Five of the seven only increased by a few percent. The other two patients increased by nearly 10%. In contrast, the remaining 28 AS patients’ gradient decreased by as much as 40% (decreasing responders).

**Conclusions:**

The orthostatic position test frequently generated a decrease of “theoretically fixed at rest” valvular gradient in AS. The combination of the stiffened stenotic valve apparatus and a reduced LV preload may be responsible for this decreasing response.

## Introduction

Recently several studies have reported the importance of upright posture during exercise in the detection of provocable (latent) or labile left ventricular outflow tract gradients (LVOTG) in hypertrophic cardiomyopathy or other obstructing conditions [1-9]. Importantly, dynamic and also passive orthostatic tests are helpful in other diseases predisposing to LVOTG [5,10]. In hypertrophic cardiomyopathy, verification of LVOTG in the upright position prior to exercise is essential. In some studies, rapid increase of LVOTG was reported only after passive orthostatic stress tests [5,7]; and in such circumstances, exercise provocation is not necessary or even contraindicated due to risk of syncope.

As far as we are aware, the orthostatic response of valvular gradients in aortic stenosis (AS) has not been performed yet. However, low-exercise (i.e. low-stress test) was proposed apart from aggressive dobutamine stimulation [11]. This examination was performed in the supine position [11]. According to the experts, exercise testing should be considered only in patients with severe AS with equivocal or no symptoms and who are eligible for aortic valve replacement [12].

The different anatomical conditions, such as flexible (muscular) subvalvular obstruction versus stiffness (atherosclerotic) valvular stenosis may induce different hemodynamic response to preload reduction in the upright position. The aim of this study was to assess the influence of the orthostatic stress test on changes in aortic pressure gradients in AS patients.

## Methods

We performed a full, standard echocardiography in the supine position. We obtained the M-mode and two-dimensional echocardiograms for each patient which was followed by a pulsed and continuous-wave Doppler ultrasound. We used conventional techniques to measure the echocardiographic parameters. The stress role in the orthostatic test was assessed in a total of 56 AS patients who did not demonstrate other serious valvular diseases. Patients were required prospectively during period of one year. We have included all AS patients without exclusion criteria as follows. 21 patients were excluded from the study for three reasons: 1) atrial fibrillation (significant beat-to-beat variability of measured gradients, 2) suboptimal Doppler signal during orthostatic test, and 3) aortic gradient significantly higher in suprasternal or right parasternal windows than in the apical window (different direction of stenosed blood jets) in the supine position. The last limitation (#3) is methodologically important because during the orthostatic examination only the transapical measurement was used. The modifying role of atrial fibrillation in AS has been recently described [13]. Ultimately, we analyzed 35 AS patients (20 males, 15 females, mean age 74.8 ± 9.2 years).

The study has been accepted by local Ethic Committee of Collegium Medicum Jagiellonian University (KBET/130/B/2011).

### Orthostatic test

We had the patient stand for 1 minute with their left hand on their head [5,8]. We made the measurement from the apical window.

Statistical analysis: Data are expressed as means ± standard deviation. The Kolmogorov-Smirnov test was used to determine normal distribution. Differences were tested for statistical significance, using paired t-test. A p value below 0.05 was considered significant.

## Results

**Figure 1:**
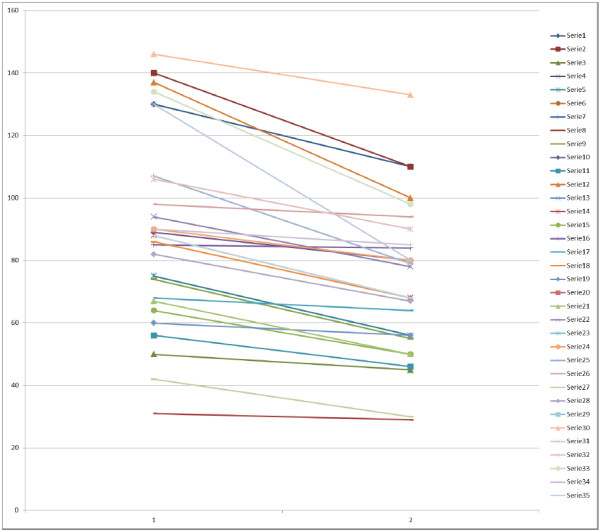
**Changes of maximal gradient in individual AS patients (1 = supine, 2 = upright).** List of abbreviations: LVOTG = left ventricular outflow tract gradient, AS = aortic stenosis.

**Figure 2:**
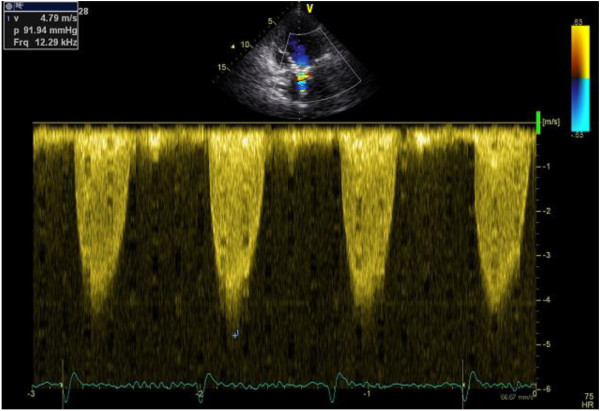
Maximal gradient in supine position.

**Figure 3:**
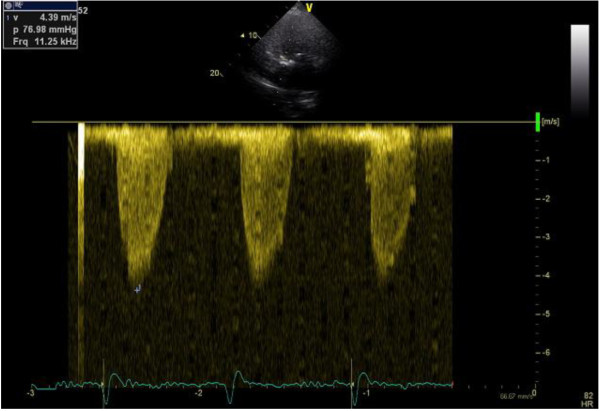
Maximal gradient in upright position (common reaction decrease about 15%).

All patients were symptomatic. The wide range of severity of AS was examined (maximum aortic gradient in the supine position from 30 to 146 mmHg). Data of individual patients were presented in Figure 1. Example of gradient measurement were displayed in Figure 2 (supine) and Figure 3 (upright) – common response with gradient decrease about 15%. The remaining echocardiographic parameters were summarized in Table 1. With regards to the statistical trends, the maximum aortic gradient significantly decreased after orthostatic stress (87.5 ± 28.6 to 75.8 ± 23.7 mmHg p > 0.01). In 7 patients (increasing responders) the peak aortic gradient slightly increased during the stress test (5 experienced only a few percent increase and in 2 by nearly 10%). These 2 patients made up our minigroup who showed positive response, in contrast to the remaining 28 AS patients whose gradient decreased up to 40% (decreasing responders).

**Table 1:** Characteristic of AS patients – baseline measurement at supine position

Maximal gradient (mm Hg)	87.5 ± 27.6
Mean gradient (mm Hg)	52.3 ± 20.1
Aortic valve area (cm2)	0.77 ± 0.22
LV end-diastolic diameter (mm)	49.1 ± 8.1
LV end-systolic diameter (mm)	31.9 ± 9.3
Septum thickness et diastole (mm)	13.6 ± 1.7
Posterior wall thickness et diastole (mm)	12.2 ± 1.4

## Discussion

With an increasing prevalence of patients with valvular heart disease, a dedicated management approach is needed [14]. The evaluation of valve defect only under resting conditions might underestimate the full impact of the lesion and its clinical effects [15]. Orthostatic-induced alterations in Doppler echocardiographic measures of ventricular function have not been well-defined and in very recent study [16] echocardiographic changes from the supine to the sitting position were measured in a small group. However, the sitting position causes less hemodynamic stress (but is more comfortable) than the standing position. Standing is a fundamental activity of daily life [1] and may induce a fall in cardiac patients predisposed to syncope due to (sub) valvular obstruction. The Orthostatic test is a provocative maneuver that is definitely physiologically based and most relevant to conditions under which patients predisposed to (pre)syncope incur symptoms. In HCM patients standing is recommended as a physiologic provocative maneuver [6-8] and in some patients standing may guide therapy. Measuring subvalvular gradients by echocardiography in the supine position, as is routine practice for assessing patients, does not reflect the pathophysiology of this pathology during daily activities, which trigger the symptoms that patients report to their cardiologists.

In the current study among AS patients, the mean value of maximum aortic gradient significantly decreases after orthostatic stress. In minority patients peak aortic gradient slightly increases during the stress test (mainly by a few percent) in contrast there is a decrease in the majority of AS patients (sometimes more than one-third). All patients were symptomatic. In previous studies with different patients i.e. asymptomatic [12,17] exercise as stress was preferred and the provocation of the gradient was measured in the (semi) supine position. This methodology facilitates the measurement of the gradient. Additionally in the supine position, the left ventricular preload is greater than in the upright position. From a methodological point of view, the standing position is more natural and reflects a more physiological condition during everyday activity than the supine position [6]. In every-day life, AS patients have experienced syncope mostly in the upright position. Using the orthostatic test, we tried to reproduce the physiological conditions occurring during daily life.

We postulate that future Guidelines of Scientific Societies should recommend the orthostatic stress test (isolated or combined with exercise) to be employed in groups of patients with predisposition/presence of subvalvular or valvular gradients.

We did not calculate LV diastolic and systolic volumes in the standing position. In the standing position, we concentrated on the gradient measurements from the apical window. However, we did not perform short and long parasternal axis imaging. Additionally, we cannot simultaneously measure both gradient and LV dimensions, and we chose to focus on gradient recording as the main aim of this study. The measurement of LV dimensions in the standing position is difficult to obtain, and the alternative stress test, i.e. lower body negative pressure (LBNP), may provide an opportunity to measure LV unloading in the supine position (the most suitable for echocardiography). However LBNP is not a natural stress test in contrast to the natural standing position which is common everyday activity. The orthostatic stress test has additional advantages. It is fast, simple, without any cost and any devices.

### Limitation of study and further direction of studies

The exclusion criteria (methodological) in the study significantly constrained patient recruitment. In the upright position, only the apical window was used for aortic gradient measurement. The preload may be the most important for gradient changes. According to our experience and data for others publications, comprehensive echocardiographic examination is hard to perform in upright position. Practically only apical view is imaginable. In previous studies in aortic stenosis the echocardiography was performed in semi-supine position. New approach (novel position) have been proposed by Rowland T, et al. [16]. Authors have performed examination in young healthy males in sitting position, however, not in upright (erect) position. In our study not only position (erect) during echo recordings was more difficult but also study group was more demanded for echo imaging. Our unselected patients were elderly (mean age 75 years), frequently obese or with pulmonary emphysema. The group consisted of many women with breast responsible for additional difficulties in imaging. The group represent “natural AS patients”. Rowland et al. studied only very young, male, non-obese patients.

In comparison to our previous study in hypertrophic cardiomyopathy [5], the LVOT gradient is easier to measure than the transvalvular gradient via the stenosed aortic valve. Consequently, we did not extend our pilot study to the upright exercise test. In the standing position, the low-exercise fixed load may be the more advantageous test [11]. However, it should also be considered that the transvalvular gradients measured by the Doppler echocardiography are highly dependent on the angle between the ultrasound beam and the flow jet direction through the narrowed valve, which may vary during exercise. Importantly, the assessment of AS by exercise-stress echocardiography is technically challenging even in experienced hands and time consuming, which may limit its application in clinical practice. It should be added that exercise Doppler echocardiography can only be performed in patients with adequate echocardiographic windows [11].

Probably in a substantial portion of patients, transvalvular thin blood jet should be hard to measure during patient movement and breathing motion. However, such measurements at peak exercise may be particularly informative for patients with presyncopal episodes during exercise. In our AS group, a minority of patients experienced increased gradients. The number of ‘non-dippers’ was too small for statistical comparison but we expect that this subgroup may have more preserved LV contractility and may be candidates for valve repair later than the decreasing responders group. Further studies with a larger group of patients is needed to verify the hypothesis about the role of contractility force and depressed load.

We preferred the simple upright test than the tilt table test. We simply achieved “true stress”, i.e. 90 degree position (identical to every day activity). The stress was truly natural without any cost and devices (tilt-table). It is proposed that this test is fully standardized by life conditions.

### Clinical implication

The potential role of the orthostatic test should be used in patients with borderline SA for valve replacement therapy and for monitoring procedure in post-operative patients with abnormal gradient across valvular prosthesis (borderline mismatch).

## Conclusions

In a majority of AS patients the transvalvular gradient decreased after standing. Generally, the easy, fast and costless orthostatic stress test may be helpful in the assessment of AS patients especially those predisposed to syncope due to hemodynamic disturbances.

## Consent

Written informed consent was obtained from the patients for publication of this report and any accompanying images.

## Competing interests

The authors declare that they have no competing interests.

## Authors’ contributions

PPD design of the study, wrote and reviewed the manuscript. DS: collected data, reviewed the manuscript. All authors read and approved the final version of manuscript.
